# Monthly river temperature trends across the US confound annual changes

**DOI:** 10.1088/1748-9326/ac2289

**Published:** 2021-09-16

**Authors:** Christa A Kelleher, Heather E Golden, Stacey A Archfield

**Affiliations:** 1 Department of Earth and Environmental Sciences, Syracuse University, Syracuse, NY, United States of America; 2 Office of Research and Development, US Environmental Protection Agency, Cincinnati, OH, United States of America; 3 Water Resources Mission Area, US Geological Survey, Reston, VA, United States of America

**Keywords:** trend analysis, water quality, stream temperature, regulation, water management

## Abstract

Climate variations and human modifications of the water cycle continue to alter the Earth’s surface water and energy exchanges. It is therefore critical to ascertain how these changes impact water quality and aquatic ecosystem habitat metrics such as river temperatures. Though river temperature trend analyses exist in the literature, studies on seasonal trends in river temperatures across large spatial extents, e.g. the contiguous United States (US), are limited. As we show through both annual and monthly trend analyses for 20 year (*n* = 138 sites) and 40 year (*n* = 40 sites) periods, annual temperature trends across the US mask extensive monthly variability. While most sites exhibited annual warming trends, these annual trends obscured sub-annual cooling trends at many sites. Monthly trend anomalies were spatially organized, with persistent regional patterns at both reference and human-impacted sites. The largest warming and cooling anomalies happened at human impacted sites and during summer months. Though our analysis points to coherence in trends as well as the overall impact of human activity in driving these patterns, we did not investigate the impact of river temperature observation accuracy on reported trends, an area needed for future work. Overall, these patterns emphasize the need to consider sub-annual behavior when managing the ecological impacts of river temperature throughout lotic networks.

## 1. Introduction

Changing climate, land cover, and water management approaches are fundamentally reshaping pathways of water and heat exchanges around the globe (Jimenez Cisneros et al 2014, Condon et al 2020). Land-based, global mean surface temperatures are warming at historically unprecedented rates (IPCC 2014). Likewise, impervious surface expansions in urban areas (Sleeter et al 2013) are altering the timing and rate of water delivery to urban waters (Bhaskar et al 2020), and reducing riparian shading, which increases incoming solar radiation at the air–water interface (Dugdale et al 2018). Water management, including irrigation, dams, and canals, also considerably alters riverine water and heat exchanges (Cai et al 2018). Altogether, these changes are modifying water temperatures now and likely into the future (van Vliet et al 2013, Hannah and Garner 2015, Jackson et al 2018).

Data-driven and model-based studies examining historical river temperature trends around the world demonstrate that riverine river temperatures are warming (Webb 1996, Kaushal et al 2010, Islam et al 2019), though regional cooling has also been observed to a lesser extent (Arismendi et al 2014). Many studies have focused on annual trends. However, temperature-related impacts to hydrology and meteorology—and therefore the aquatic ecosystem—are likely to occur at seasonal and sub-seasonal scales. Quantifying sub-annual trends in river temperatures is particularly important given the strong connections between river temperatures and other sub-annual aquatic dynamics, including stream metabolism, dissolved oxygen reductions, and stream greenhouse gas production (Appling et al 2018, Comer-Warner et al 2018, Song et al 2018). The propagation and mitigation of these impacts demands further investigation of how river temperature trends manifest throughout the year, as this information is necessary to identify likely drivers of these changes.

To address this, we conducted novel monthly trend analyses of river temperatures across the United States (US), comparing monthly results to average annual trends. Overall, we expected the pattern of monthly river temperature trends to mirror the patterns of annual river temperature and air temperature trends across the US. Currently unknown is to what extent seasonal trends in river temperatures are impacted by regulation controls affecting river flow or are organized by physiographic region. We expected monthly trends to be of similar direction (warming/cooling) within physiographic regions except for sites impacted by regulation controls. Our findings provide new insights into sub-annual variations in river temperature trends across a gradient of physiographic regions and human impacts, and the spatial discretization necessary to understand them.

## 2. Methods

### 2.1. River and air temperatures

**Figure 1.**
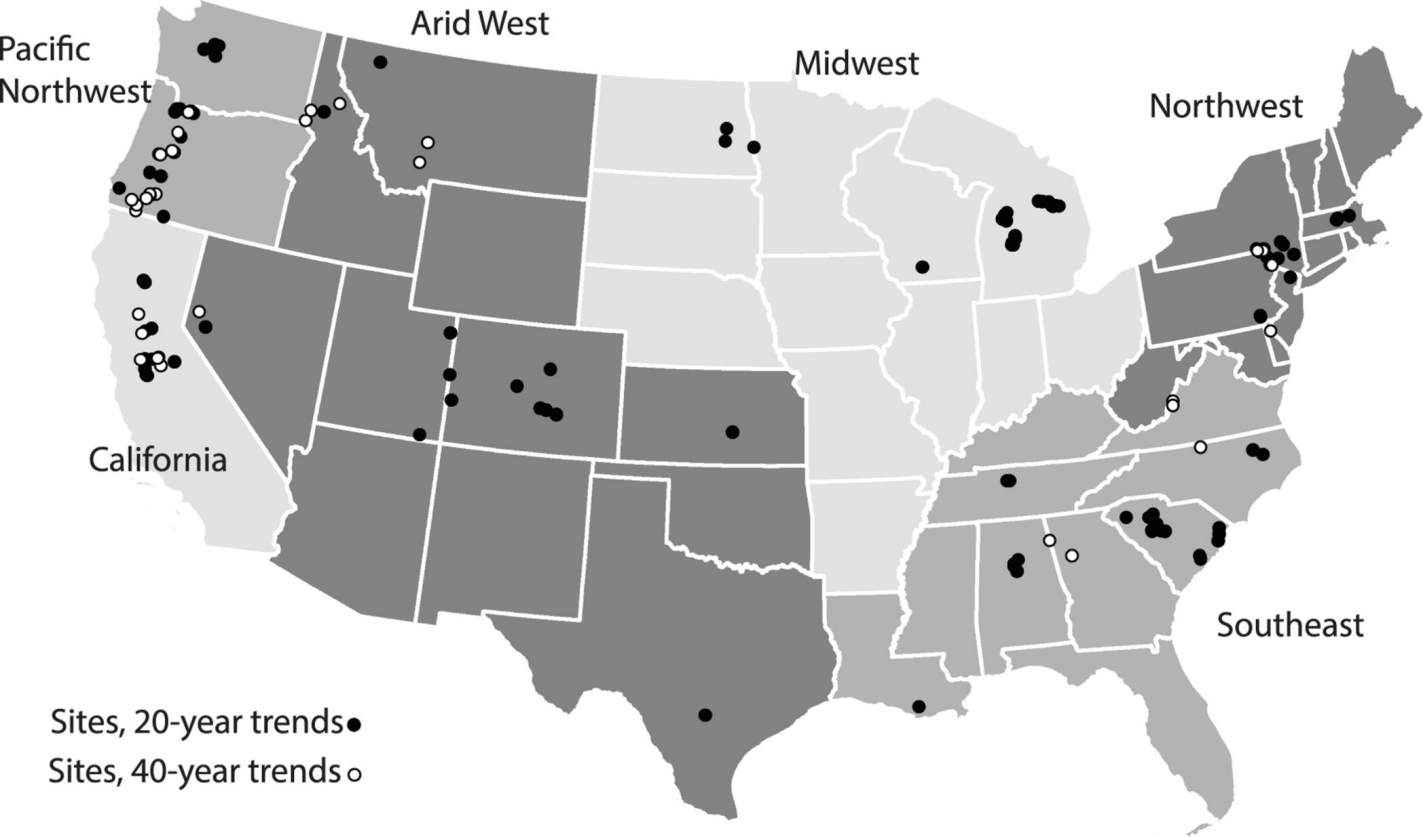
Sites assessed for 20 year and 40 year trends in river temperature. Regions shown in this figure are used for grouping and analyzing results (see figures 2 and 3).

We analyzed trends in monthly-averaged daily maximum and daily minimum river temperature across a 40 year period (1979–2018) and a 20 year period (1999–2018). We assessed maximum and minimum river temperatures, as studies have observed changes to nighttime temperatures are outpacing changes to daytime temperatures (Davy et al 2017). We used the US Geological Survey (USGS) National Water Information System to identify sites with at least 20 years of daily river temperature values, from 1999 through 2018 (United States Geological Survey 2020). Through this process, we identified 138 sites with at least 20 years of observations and 40 sites with at least 40 years of observations (figure 1). Sites were selected to contain no more than 2 years of missing data for 20 year trends and no more than 6 years of missing data for 40 year trends. There were 35 common sites between the two trend periods, as some of the 40 year sites did not meet the more rigorous missing data criteria for 20 year trends. Study sites span the contiguous US and include sites that vary in terms of drainage area (minimum: 13 km^2^, median: 2545 km^2^, maximum: 240 662 km^2^) and human impacts (figure 1).

We extracted daily values of maximum and minimum river temperature using the data retrieval package (De Cicco et al 2018) in R (v1.2.5) and averaged them to months. Information on the procedures used by the USGS to generate accurate river temperature measurements can be found in Wilde (2006). As time series for river temperature are notoriously incomplete, we treated months missing more than 50% of daily observations as a missing data value in the analysis. Approximately 129 (of 138) sites were missing less than 12 months of data for analyzing 20 year trends and 28 (of 40) sites were missing fewer than 36 months of data for 40 year trends. Monthly averaged daily maximum and daily minimum air temperature values were also extracted at all relevant sites using the parameter-elevation regressions on independent slopes model (PRISM; PRISM Climate Group 2004, Daly et al 2008). Data was accessed using the R PRISM package (v0.1.0) using R software for all sites (Edmund et al 2020).

### 2.2. Site classification

We classified sites to enable interpretation of trends in the context of dams and streamflow regulation, urbanization, and agriculture. Each site was classified into one of seven different categories:

- Reference sites: sites with limited human impact, as defined by previous work by Falcone et al (2010) and Falcone (2011).
- Proximally regulated sites: those within 5 km downstream of a major dam (height of 15 m or greater, or total storage of approximately 6 millionm^3^ or greater; Falcone et al 2010, Falcone 2011) regardless of surrounding land cover.
- Regulated sites: those with an upstream dam (but no proximal major dam) and limited developed (<10%) and agricultural (<10%) land cover.
- Compound stressor sites: those with an upstream dam (but no proximal major dam) combined with upstream developed (>10%) or agricultural (>10%) land cover.
- Agri-urban sites: those with no dam upstream but >20% agricultural and >20% urban land cover.
- Agricultural sites: those with no dam upstream but >20% agricultural land cover.
- Unclassified sites: sites that were not able to be classified with available information.

At 100 sites, information was drawn from the GAGES II dataset (Falcone et al 2010, Falcone 2011). For these sites, we extracted several calculated metrics, including the distance to nearest dam, distance to nearest major dam, presence of upstream dams, and the percentage of developed, forested, and agricultural land cover. The remaining sites were classified by first delineating each watershed in StreamStats (United States Geological Survey 2021), calculating the distance to the nearest upstream major dam within the National Dams Inventory (United States Army Corps 2019), and extracting developed, agricultural, and forested land cover percentages (Multi-Resolution Land Characteristics Consortium 2020). StreamStats delineation was not possible for a few sites; in these cases, we identified USGS gages within the GAGES II dataset that are proximal to these sites. We were able to classify all but one site. These classifications are summarized in table S1 (available online at stacks.iop.org/ERL/16/104006/mmedia), with additional classification scheme details described in text S1.

### 2.3. Statistical analysis

We conducted trend analysis using the nonparametric seasonal Mann–Kendall (seasonal MK) trend test (Hirsch et al 1982). The seasonal MK trend test is a variation of the original MK trend test (Mann 1945) that computes the MK test for each season, and then combines the results to obtain a measure of annual trend significance, direction, and overall strength of the trend (Helsel et al 2020). In our application, a season is equal to 1 month. If strong positive (warming) and negative (cooling) seasonal trends exist in the series, it is possible that these seasonal trends will cancel one another out when aggregating to the annual test statistic. Therefore, little to no trend will be detected at the annual scale, masking important underlying seasonal trends (Helsel et al 2020). As a result, we analyzed both the seasonal MK annual trend results and the trend results for each individual season (i.e. month) (Helsel et al 2020).

We report trends for monthly and annual maximum and minimum river temperatures for a 40 year period, from 1979 to 2018 (*n* = 40) and a 20 year period, from 1999 to 2018 (*n* = 138). For sites with 40 years of river temperature observations (*n* = 40), we also calculated annual and monthly trends for each year from 20 (1999) to 40 years (1979), to investigate how trends change with record length. Trend significance (*p*-value) is expressed as the probability that the test statistic of the seasonal MK is not significantly different from zero. The strength of the correlation between time and temperature is measured by tau, a measure of correlation that ranges between −1 and 1. Due to the mechanics of its calculation, lower values of tau are equivalent in strength to higher values of the commonly-used Pearson *r* for linear correlations (Helsel et al 2020). Trend magnitudes are reported as the Sen slope (also known as the Thiel–Sen slope; Helsel et al 2020) in degrees per decade. Trend assessment was performed in Matlab (R2019b; Burkey 2020a, 2020b).

As of 2018, daily river temperature resolution has been 0.1 °C at all sites; however, resolution varied across sites and time given ongoing improvements in sensor technology. To assess whether this shift in resolution impacted annual and monthly trends, we examined a subset of sites (*n* = 57, 20 years; *n* = 11, 40 years) for which daily river temperatures were reported consistently at 0.1 °C resolution. For these sites, we rounded daily river temperatures to the nearest 0.5 °C for 1979 through 2007 (40 years) or 1999 through 2007 (20 years), and recomputed all trends. The year 2007 was selected as nearly all sites (87.5%, 40 years; 93.6%, 20 years) contain daily river temperatures at 0.1 °C from this year forward. While the resolution of these observations implies a known accuracy (and shift in accuracy through time), we did not investigate the impact of this accuracy on reported trends.

To explore potential patterns between trends and watershed characteristics, we statistically compared river temperature trends to site drainage area, latitude, and trends in air temperature (assessed using the same methods for river temperature trends). River temperature trends were aggregated regionally (following groupings shown in figure 1) and as a function of site classification (see table S1). Trend distributions aggregated by watershed drainage area were compared to assess for statistical similarity or difference using the two-sample Kolmogorov–Smirnov test.

## 3. Results and discussion

### 3.1. Similarities and differences amongst annual and seasonal trends

**Figure 2.**
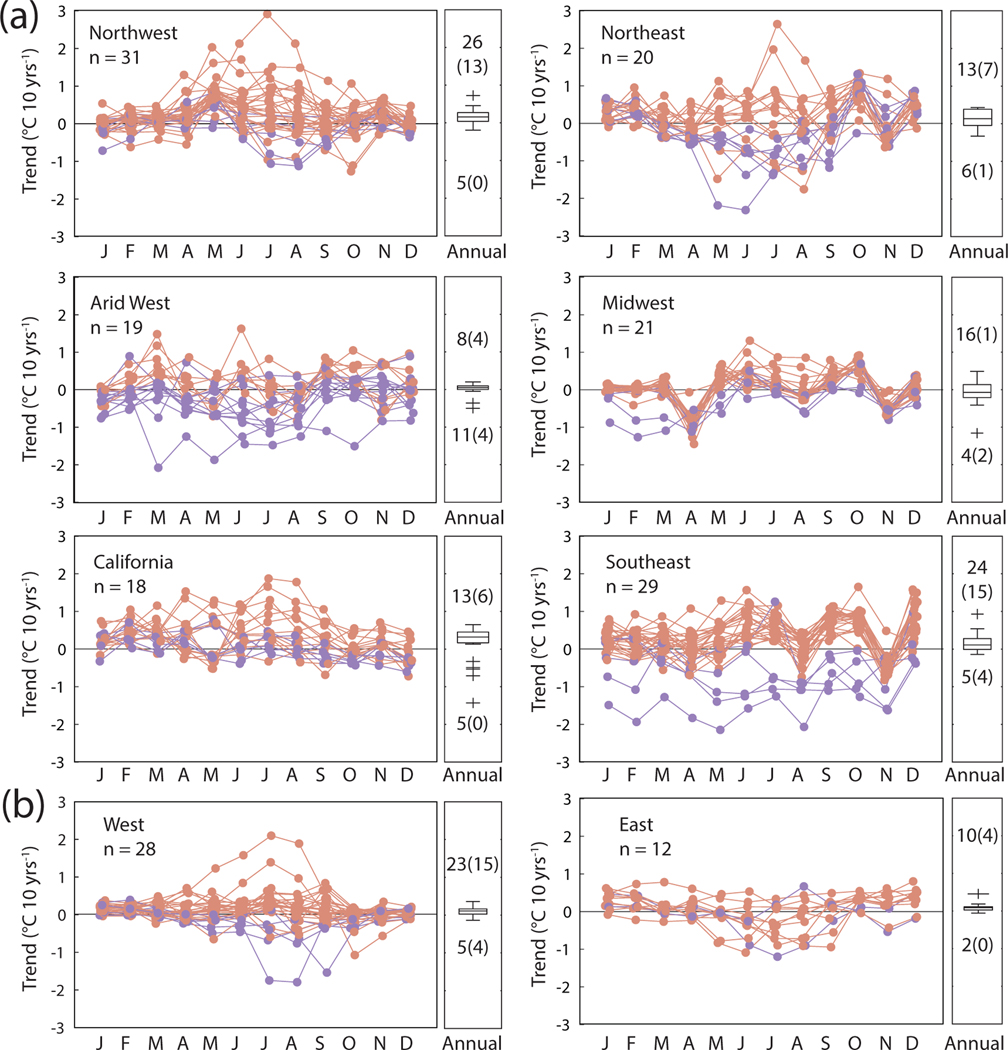
Regional annual and monthly river temperature trends. (A) Annual trends assessed via the MK trend test and monthly 20 year trends assessed via the seasonal MK trend test for monthly averaged daily maximum river temperature shown for six regions. (B) Annual and monthly 40 year trends are shown for eastern and western sites. Monthly trends are shown as line plots, while annual trends are aggregated into box plots (on right-side of panels). Line colors indicate whether annual trends are positive (red) or negative (blue). Numbers within boxplots indicate the number of positive and negative trends, excluding trends equal to zero. Numbers in boxplot parentheses indicate the number of trends that were significant (*p* < 0.05). Note two sites had annual trends equal to zero. Results for monthly-averaged daily minimum river temperatures are shown in figure S1.

Both significant and non-significant trends in maximum and minimum river temperature across 138 sites (20 years of record) and 40 sites (40 years of record) overwhelmingly indicated annual warming (figure 2; figure S1). This was true at 72% (daily maximum) and 86% (daily minimum) of sites for 20 year trends, and 83% (daily maximum) and 88% (daily minimum) of sites for 40 year trends. We report tau values in table S3. Annual trends across a subset of sites with long term data were generally static through time (figure S2). Our measurement accuracy test confirmed similar annual and monthly trend results when coarsening data from 0.1 °C resolution to 0.5 °C resolution (figures S3 and S4).

Annual 20 year trends were significant (*p*-value <0.05) at more than 40% of sites for maximum and minimum river temperature observations (table S2). The majority of these significant trends were positive (81%, maximum; 94% minimum). For 40 year trends, 58% (maximum) and 68% (minimum) of trends were significant. Again, a large percentage of these significant trends were positive (83% maximum; 89% minimum). The percentages of sites with significant warming and cooling trends mirror the percentages found for the entire dataset.

**Figure 3.**
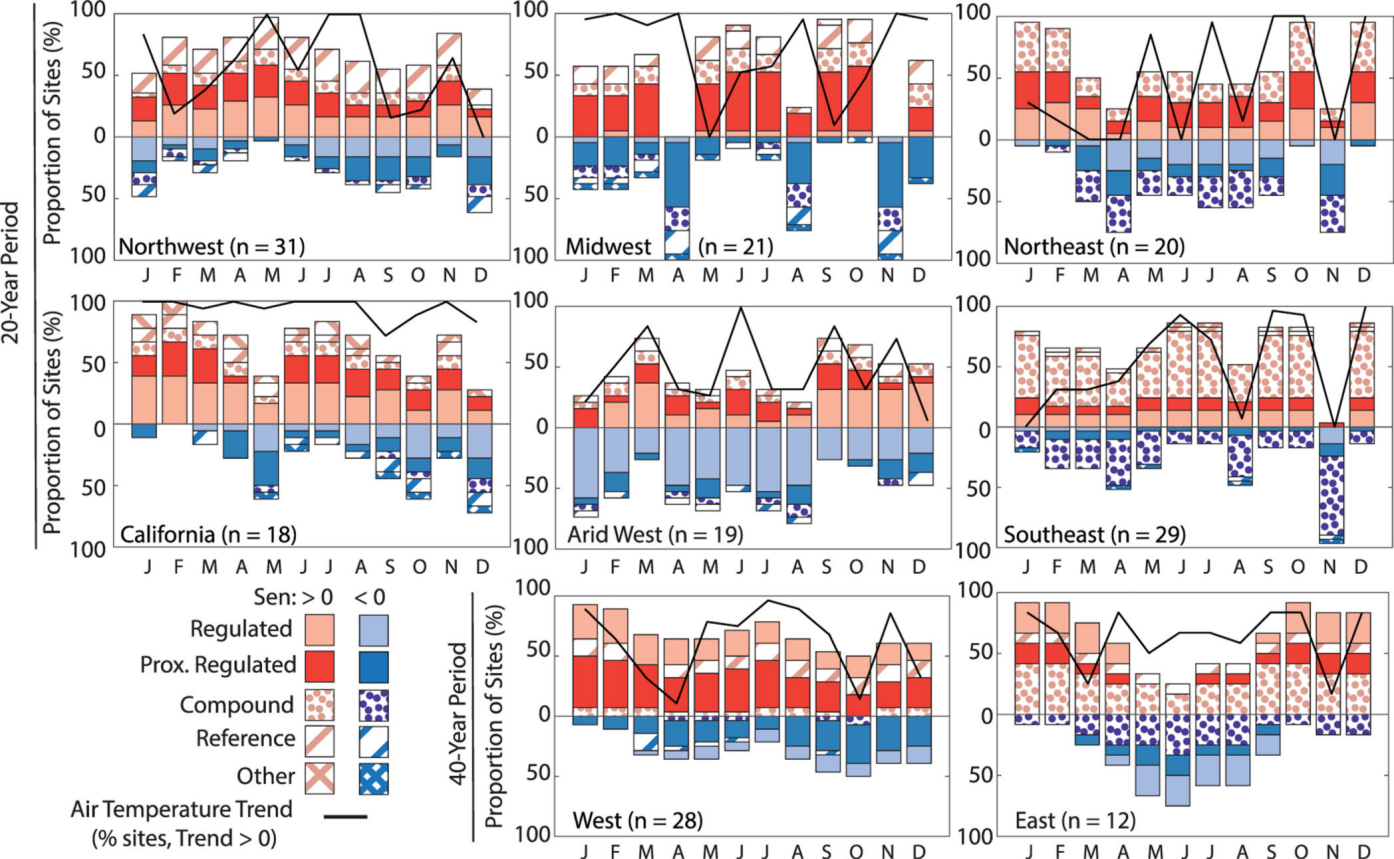
River temperature trend signs organized by region and human impact. Monthly 20 year (top) and 40 year (bottom) trends for monthly-averaged daily maximum river temperatures (determined via MK) showing the number of sites with positive (Sen > 0) or negative (Sen < 0) trends. While 20 year trends span the conterminous US (figure 1), 40 year trends were only concentrated in the west and eastern US. Black lines indicate the proportion of sites with positive monthly air temperature trends. Fill indicates site classification. Results for monthly-averaged minimum river temperature trends are shown in figure S5. Class ‘other’ compasses one agri-urban site, two agriculture sites, and one unclassified site.

Twenty-year trends revealed warming river temperatures in most seasons (figure 3; tables S4 and S5). In aggregate, warming trends in monthly-averaged daily maximum river temperatures were observed at more than 50% of sites in all but 3 months (April, August, and November). The same was true for monthly trends in daily minimum river temperatures except in November. For each month, more than 50% of sites displayed positive monthly 40 year trends (table S6). One caveat to our analysis is that many sites reach or approach 0 °C during winter months. These sites are problematic for annual and monthly trend analysis because they are more likely to warm than to cool. For instance, many sites display positive trends during winter months, and especially for the 40 years trend period (figure 3). Regardless, these findings are in line with several studies from the US and around the world that have shown historical annual and monthly river temperatures have warmed over the recent past (Kaushal et al 2010, Hannah and Garner 2015, Michel et al 2020, Hare et al 2021).

While aggregated river temperature trends suggest annual and monthly warming is the dominant pattern across all sites, this relationship did not hold when examining the persistence of warming trends at individual sites. Few sites unilaterally warmed across all seasons (figures 2 and S1, table S7). For example, 20 year trends in monthly-averaged maximum river temperatures increased in all seasons at only two sites. Likewise, only six sites were found to be warming in all months for monthly-averaged minimum river temperature trends. While 40 year trends examined a smaller number of sites (*n* = 40), only two (daily maximum) and three sites (daily minimum) displayed warming trends across all months (figure 3; table S8).

Magnitudes of annual river temperature trends were muted compared to those of monthly warming and cooling trends (figure 2). For example, more than 90% of sites registered annual 20 year trends between ±0.5 °C dec^−1^, yet monthly-averaged maximum and river temperature trends exceeded 0.5 °C dec^−1^ and fell below −0.5 °C dec^−1^ in at least 1 month at more than 90% of sites. Large magnitude cooling trends were especially pronounced in early spring (April) and late fall (November), and large magnitude warming trends commonly occurred in summer and fall months. While probability would dictate annual trends are muted compared to seasonal responses, these US-wide results underscore the importance of managing water quality impacts at both annual and seasonal scales.

### 3.2. Seasonal trends: patterns and anomalies

Persistent patterns in warming and cooling were observed for nearly all regions for 20 year trends. Though containing only 18 sites, we note that river temperature cooling in 20 year trends occurred for a majority of CA sites in May and December. Likewise, across a small number of sites in the Arid West (*n* = 19), cooling trends for the 20 year period were observed during winter and spring months (excepting March). This is notable because these regions typically receive the majority of annual precipitation during winter months, with May (depending on site elevation) often corresponding to the spring snowmelt season. In reference rivers, there is potential that shifting the timing of snowmelt may alter the timing and temperature of water feeding streamflow (Yan et al 2021). In regulated areas, dam operation likely reflects this shift in inter-annual conditions (Steimke et al 2018). River temperature cooling trends persisted in April, August, and November in the Midwest, April and November in the Northeast, and November in the Southeast. The majority of 40 year monthly trends were positive, though summer cooling persisted during summer at sites in the eastern US (figure 3).

**Figure 4.**
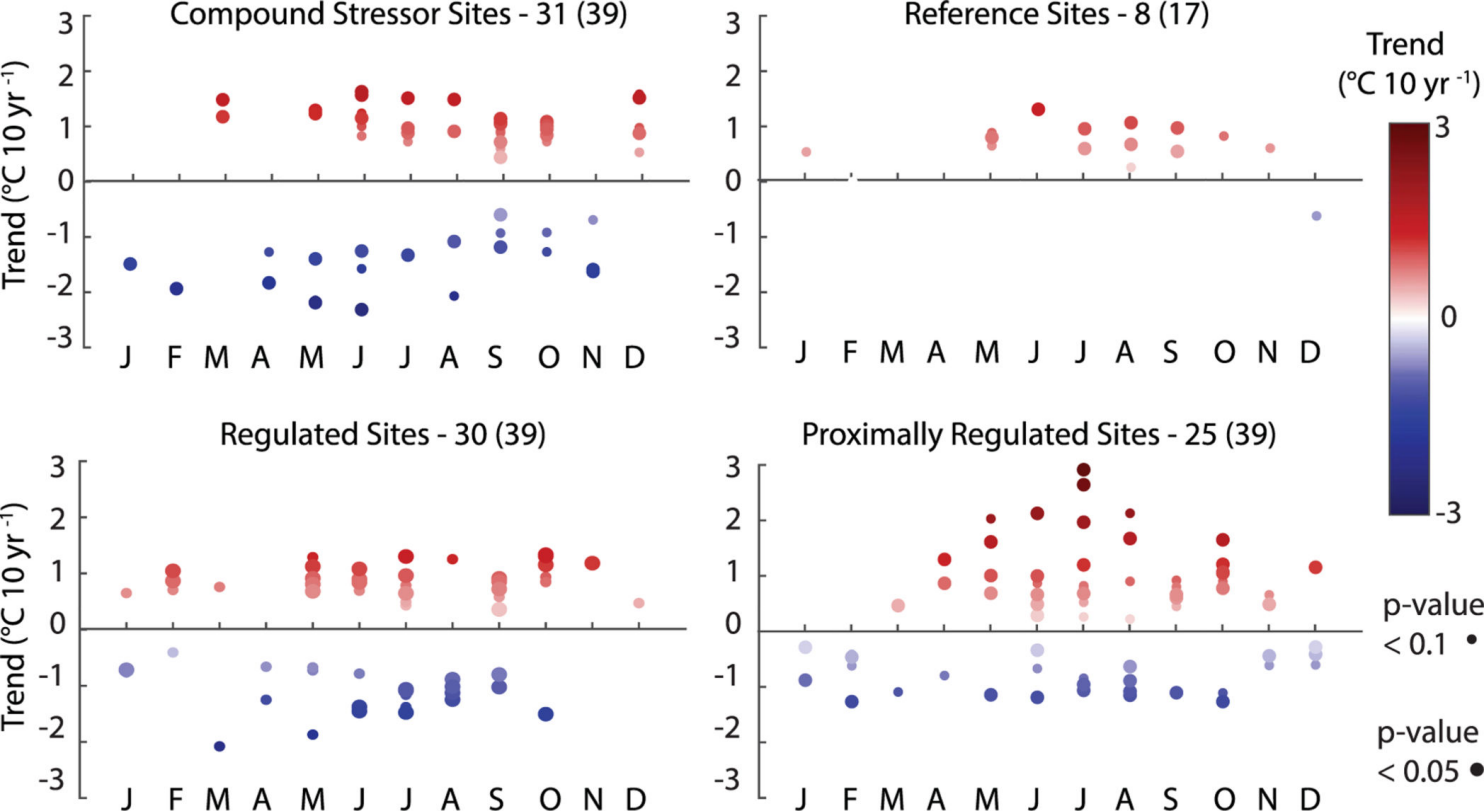
Statistically significant monthly, 20 year Sen slopes organized by site class. Sen slopes are shown for monthly-averaged daily maximum river temperatures. We report the number of sites with statistically-significant trends during any month, with the total number of sites within each class shown next to this number in parentheses. Results for four sites (one agri-urban site, two agriculture sites, one unclassified site) were omitted from the figure.

We observed two primary anomalous patterns in seasonal river temperature trends across sites and regions: large magnitude anomalies that were unique to individual sites (figure 2), and anomalies (i.e. large magnitude deviations from zero trend) that were associated with individual sites (figures 3 and S5). For example, while we found general patterns in the direction of trend, many sites uniquely exhibited statistically significant and large magnitude cooling or warming trends for the 20 year record and 40 year record within different regions (figure 4; table S5). The majority of statistically significant trends occurred between late spring and mid-fall and were largest during the summer months for both the 20 year and 40 year record.

Statistically significant anomalies tended to occur at human-impacted sites as opposed to reference sites (figure 4). Across these sites, we hypothesize that the major driver of such anomalous warming and cooling trends is likely streamflow regulation. The mere presence of upstream regulation would not generate a trend; instead, this suggests that changes to dam operation—either as a function of the magnitude and timing of water release or location of water release—are likely generating the observed trends. As reservoirs are thermally stratified, the location of dam release may either be hypolimnetic (cold) or epilimnetic (warm) (Olden and Naiman 2010). Additional complexity is introduced because many of these river systems contain multiple dams (e.g. O’Keeffe et al 1990) and experience additional influence from upstream tributaries. Finally, the impact of upstream regulation cannot be truly regarded as independent from climate and weather, as both short-term (a week or less) and seasonal weather forecasts (weeks to months) are regularly used in reservoir operations (Block 2011, Nayak et al 2018).

### 3.3. River temperature versus air temperature trends

Trends in river temperature are often linked to trends in air temperature, as net solar radiation is a major driver of land surface and river temperatures (Caissie 2006). However, our results demonstrate that while several regions showed apparent correspondence between air temperature and river temperature trends (figure 3), monthly river temperature trend magnitudes did not closely mirror those of air temperatures in most instances. For example, sites in CA and the Midwest displayed deviation from these patterns. In these regions, reference sites were rare, and nearly all sites are classified as impacted by regulation or regulation combined with other land cover impacts.

We found that 20 year trends in river temperature regularly exceeded trends in air temperature, demonstrating that river temperatures are warming at greater rates than air temperatures in many seasons (table S8). We do note that trends in air temperature are expected to deviate from trends in river temperature during winter months for sites with river temperatures at or near 0 °C; in these locations and under these conditions, air and water temperatures are disconnected (Mohseni et al 1998). During summer (June–August), trends in river temperatures (both maximum and minimum) both exceeded or fell below air temperature trend magnitudes, depending on location.

The finding that trends in air and water temperatures differ in magnitude is in many ways unsurprising, as numerous studies have established that the slope of relationships between monthly and annual air and water temperatures are rarely one-to-one (Webb and Nobilis 1997, Erickson and Stefan 2000) and that relationships between air and water temperatures are nonstationary through time (Arismendi et al 2014). The strength of the air–river temperature relationship is modified by a host of site-specific factors, including riparian shading, groundwater buffering, and upstream regulation, among others, as well as evaporation that can cool warm water temperatures as air temperatures continue to rise (Mohseni et al 1998). However, our findings suggest that warming in rivers is outpacing warming at the land surface (vis-à-vis air temperature trends) during unexpected periods of the year—winter, spring, and fall. The differences between the behavior of air temperature and river temperatures may be attributed to the effect of multiple interactive, short-term dynamics superimposed onto longer term variations in streamflow (as well as sources of water to the stream) and human modifications to the upstream or riparian environment. These processes reflect the propagation of a changing climate and other human impacts on the magnitude and timing of hydrologic processes, which thereby impart complex energy exchanges along rivers (Poole and Berman 2001).

### 3.4. Spatial organization, river size, and human impacts

How do we discern important drivers of the trends we present? This overarching goal of all trend studies remains a challenge. Annual river temperature trend magnitudes at both 20 year and 40 year periods did not appear to be spatially organized (figures S6 and S7), and displayed no strong linear or nonlinear relationships with latitude or drainage area (tables S9 and S10). However, distributions of sites with low (<100 km^2^) and moderate (100–2000 km^2^) drainage areas had higher average magnitude trends than those of large drainage area sites (>2000 km^2^; figure S8). The distributions of trends at sites with small drainage areas versus large drainage areas were statistically significantly different (according to the two-sample Kolmogorov–Smirnov test with a 5% significance level). This empirically suggests that large rivers may be less impacted by climate change than small or moderately sized rivers, or that flow regulations or multiple human impacts, which commonly occur and compound on large rivers, are modulating this warming. This is supported by work that has shown variance in river temperatures (as well as streamflow) are dampened for increasing drainage areas (Caissie 2006). However, given the limited coverage of our sites, more work is needed before this can be definitively concluded. In addition, ascribing causes of such change, in terms of trend magnitude and significance, is challenged by the accuracy of stream temperature datasets. We caution that, due to limited information, our analysis did not investigate the impact of river temperature observation accuracy on annual and monthly trends. The USGS is known to produce data of exceptional quality, though accuracy of observations has likely increased through time, with unknown implications for the trends reported herein.

While human modification of the water cycle is likely to have strong cascading impacts on long-term water quality, we show that distinguishing drivers of these trends can be challenging. Regardless of classification, trends support a common message regarding the response of river temperatures to changing climate and other associated impacts. When trends were organized regionally, patterns in warming and cooling persisted regardless of the type of human impact (figures 3 and S5). This suggests that while magnitudes of trends may differ at regional to national scales, rivers are likely warming and cooling in response to similar drivers (i.e. weather and climate, modulated by reach and watershed form and water management). Likewise, though few of our sites fell into the reference category (*n* = 17, 20 years; *n* = 5, 40 years), reference sites tended to have fewer statistically-significant seasonal trend anomalies and lower magnitude trends as compared to human-impacted sites (figure 4).

Given that a majority of sites with long-term temperature data for analysis are human-impacted, located along large rivers, or both, it is likely that observed trends are a composite of changing climate mixed with human modification of the water cycle, including flow regulation, forest loss, the growing presence of impervious surfaces, varying water withdrawals, changing magnitudes and temperatures of hydrological exchange flows along the river network, and removal of riparian vegetation (Lessard and Hayes 2003, Drummond and Loveland 2010). Many of the anthropogenically impacted sites within our analysis had already experienced modification by 1970, the start of our trend analysis period. Climate change may also impart diverse effects on river temperatures indirectly via its influences on aquatic energy exchanges, including shifting the timing and magnitude of snowmelt (Dudley et al 2017), changing fractions of precipitation falling as rain versus snow (Berghuijs et al 2014), increasing the frequency of floods, droughts, and heatwaves (Armstrong et al 2012, Dai 2013), and changing the frequency and magnitude of precipitation (Mallakpour and Villarini 2017). Further, across long time scales (multiple decades), climate teleconnections can impact river temperature trends (Islam et al 2019).

The potentially large number of interacting drivers affecting river temperature trends spell unclear outcomes regarding the magnitude of changes into the future. Research is needed to quantitatively discern these diverse drivers across sites and especially within reference settings (e.g. Arismendi et al 2012), as the patterns we and others have detected are the complex result of multiple competing factors that may sometimes augment and other times dampen impacts on river water quality.

## 4. Conclusions

Our findings corroborate that river temperatures in rivers are warming at annual timescales within the US (e.g. Kaushal et al 2010) and add to global documentation of river warming (Hannah and Garner 2015, Michel et al 2020). However, our results uniquely emphasize that focusing on annual trends in river temperature warming alone may obscure complex sub-annual warming and cooling variations occurring in rivers across the US. Given the strong association between water temperature and other measures of water quality, monthly river temperature trends are likely to confer sub-annual shifts in other water quality signals and aquatic ecosystem health undetected by annual analyses.

Around the world, rivers are expected to warm under future climate change (van Vliet et al 2013, Ficklin et al 2014). For this reason, identifying locations in the landscape that are prone to warming over long (e.g. decadal) and short (e.g. heatwaves; Piccolroaz et al 2018) timescales will be of key importance. For example, mountain environments may be buffered against changing climate, potentially because of microclimates in dense riparian cover, contributions from groundwater, and both direct and indirect impacts from snowfields (Isaak et al 2016). However, streams with shallow groundwater signatures are showing higher proportions of warming than those with deeper groundwater signatures presumably due to buffering from these and other factors (Hare et al 2021).

Given data-driven analyses may inaccurately estimate responses of rivers to climate warming (Arismendi et al 2014, Leach and Moore 2019), mechanistic modeling approaches that account for energy exchange alongside hydrological processes are needed to enable future predictions (Dugdale et al 2017). Recent developments in modeling capabilities at global scales are making such simulations possible along larger rivers (Wanders et al 2019), though more work is needed to understand how river temperatures in smaller watersheds will respond, given the complex heat budgets in these areas. Overall, these uncertainties suggest that process-based insight is needed for hindcasting and projecting future outcomes. Such insights will help to identify how complex interactions in the subsurface, at Earth’s surface, and in Earth’s atmosphere can be parsed to interpret historical trends and to predict likely impacts on future river temperatures.

## Supplementary Material

SI

## Data Availability

All data and trend analysis codes are publicly available. The data that support the findings of this study are openly available at the following URL/DOI: https://doi.org/10.4211/hs.b3773518e38b454780492d79e3a3f050 (Kelleher et al 2021).

## References

[R1] Appling AP 2018 The metabolic regimes of 356 rivers in the United States Sci. Data 5 180292 10.1038/sdata.2018.292 PMC6289110 30532078

[R2] Arismendi I, Johnson SL, Dunham JB, Haggerty R and Hockman-Wert D 2012 The paradox of cooling streams in a warming world: regional climate trends do not parallel variable local trends in stream temperature in the Pacific Continental United States Geophys. Res. Lett 39

[R3] Arismendi I, Safeeq M, Dunham JB and Johnson SL 2014 Can air temperature be used to project influences of climate change on stream temperature? Environ. Res. Lett 9 084015

[R4] Armstrong WH, Collins MJ and Snyder NP 2012 Increased frequency of low-magnitude floods in new England J. Am. Water Resour. Assoc 48 306–20

[R5] Berghuijs WR, Woods RA and Hrachowitz M 2014 A precipitation shift from snow towards rain leads to a decrease in streamflow Nat. Clim. Change 4 583–6

[R6] Bhaskar AS, Hopkins KG, Smith BK, Stephens TA and Miller AJ 2020 Hydrologic signals and surprises in US streamflow records during urbanization Water Resour. Res 56 e2019WR027039

[R7] Block P 2011 Tailoring seasonal climate forecasts for hydropower operations Hydrol. Earth Syst. Sci 15 1355–68

[R8] Burkey J 2020a Mann-Kendall Tau-b with Sen’s Method (enhanced) (available at: www.mathworks.com/matlabcentral/fileexchange/11190-mann-kendall-tau-b-with-s-en-smethod-enhanced (Retrieved 25 January 2020))

[R9] Burkey J 2020b Seasonal Kendall Test with Slope for Serial Dependent Data (available at: www.mathworks.com/matlabcentral/fileexchange/22389-seasonal-kendall-testwith-slope-for-serial-dependent-data (Retrieved 25 January 2020))

[R10] Cai H, Piccolroaz S, Huang J, Liu Z, Liu F and Toffolon M 2018 Quantifying the impact of the three Gorges dam on the thermal dynamics of the Yangtze River Environ. Res. Lett 13 054016

[R11] Caissie D 2006 The thermal regime of rivers: a review Freshw. Biol 51 1389–406

[R12] Comer-Warner SA, Romeijn P, Gooddy DC, Ullah S, Kettridge N, Marchant B, Hannah DM and Krause S 2018 Thermal sensitivity of CO_2_ and CH_4_ emissions varies with streambed sediment properties Nat. Commun 9 2803 10.1038/s41467-018-04756-x PMC6052154 30022025

[R13] Condon LE, Atchley AL and Maxwell RM 2020 Evapotranspiration depletes groundwater under warming over the contiguous United States Nat. Commun 11 873 32054857 10.1038/s41467-020-14688-0 PMC7018770

[R14] Dai A 2013 Increasing drought under global warming in observations and models Nat. Clim. Change 3 52–58

[R15] Daly C, Halbleib M, Smith JI, Gibson WP, Doggett MK, Taylor GH, Curtis J and Pasteris PP 2008 Physiographically sensitive mapping of climatological temperature and precipitation across the conterminous United States Int. J. Climatol 28 2031–64

[R16] Davy R, Esau I, Chernokulsky A, Outten S and Zilitinkevich S 2017 Diurnal asymmetry to the observed global warming Int. J. Climatol 37 79–93

[R17] De Cicco L A, Hisrch RM, Lorenz D and Watkins D 2018 DataRetrieval (US Geological Survey) (available at: https://code.usgs.gov/water/dataRetrieval (Retrieved 3 May 2020))

[R18] Drummond MA and Loveland TR 2010 Land-use pressure and a transition to forest-cover loss in the Eastern United States BioScience 60 286–98

[R19] Dudley RW, Hodgkins GA, McHale MR, Kolian MJ and Renard B 2017 Trends in snowmelt-related streamflow timing in the conterminous United States J. Hydrol 547 208–21

[R20] Dugdale SJ, Hannah DM and Malcolm IA 2017 River temperature modelling: a review of process-based approaches and future directions Earth-Sci. Rev 175 97–113

[R21] Dugdale SJ, Malcolm IA, Kantola K and Hannah DM 2018 Stream temperature under contrasting riparian forest cover: understanding thermal dynamics and heat exchange processes Sci. Total Environ 610–611 1375–89 10.1016/j.scitotenv.2017.08.198 28851157

[R22] Edmund H, Bell K and Butler A 2020 Prism: Access Data from the Oregon State Prism Climate Project (available at: https://CRAN.R-project.org/package=prism (Retrieved 4 November 2019))

[R23] Erickson TR and Stefan HG 2000 Linear air/water temperature correlations for streams during open water periods J. Hydrol. Eng 5 317–21

[R24] Falcone JA, Carlisle DM, Wolock DM and Meador MR 2010 GAGES: a stream gage database for evaluating natural and altered flow conditions in the conterminous United States Ecology 91 621

[R25] Falcone J 2011 GAGES-II: Geospatial Attributes of Gages for Evaluating Streamflow (available at: https://water.usgs.gov/GIS/metadata/usgswrd/XML/gagesII_Sept2011.xml#stdorder (Retrieved 4 May 2020))

[R26] Ficklin DL, Barnhart BL, Knouft JH, Stewart IT, Maurer EP, Letsinger SL and Whittaker GW 2014 Climate change and stream temperature projections in the Columbia river basin: habitat implications of spatial variation in hydrologic drivers Hydrol. Earth Syst. Sci 18 4897–912

[R27] Hannah DM and Garner G 2015 River water temperature in the United Kingdom: changes over the 20th century and possible changes over the 21st century Prog. Phy. Geogr 39 68–92

[R28] Hare DK, Helton AM, Johnson ZC, Lane JW and Briggs MA 2021 Continental-scale analysis of shallow and deep groundwater contributions to streams Nat. Commun 12 1450 10.1038/s41467-021-21651-0 PMC7933412 33664258

[R29] Helsel DR, Hirsch RM, Ryberg KR, Archfield SA and Gilroy EJ 2020 Statistical Methods in Water Resources (available at: 10.3133/tm4a3 (Accessed 1 July 2020))

[R30] Hirsch RM, Slack JR and Smith RA 1982 Techniques of trend analysis for monthly water quality data Water Resour. Res 18 107–21

[R31] IPCC 2014 AR5 Climate Change 2014: Impacts, Adaptation, and Vulnerability (available at: www.ipcc.ch/report/ar5/wg2/ (Accessed 10 July 2020))

[R32] Isaak DJ, Young MK, Luce CH, Hostetler SW, Wenger SJ, Peterson EE, Hoef JMV, Groce MC, Horan DL and Nagel DE 2016 Slow climate velocities of mountain streams portend their role as refugia for cold-water biodiversity Proc. Natl Acad. Sci. USA 113 4374–9 27044091 10.1073/pnas.1522429113 PMC4843441

[R33] Islam SU, Hay RW, Déry SJ and Booth BP 2019 Modelling the impacts of climate change on riverine thermal regimes in western Canada’s largest Pacific watershed Sci. Rep 9 1–14 31388033 10.1038/s41598-019-47804-2 PMC6684650

[R34] Jackson FL, Fryer RJ, Hannah DM, Millar CP and Malcolm IA 2018 A spatio-temporal statistical model of maximum daily river temperatures to inform the management of Scotland’s Atlantic salmon rivers under climate change Sci. Total Environ 612 1543–58 28915548 10.1016/j.scitotenv.2017.09.010

[R35] Jimenez Cisneros B E, Oki T, Arnell NW, Benito G, Cogley JG, Doll P, Jiang T and Mwakalila SS 2014 Freshwater Resources (Cambridge: Cambridge University Press) pp 229–69

[R36] Climate Change 2014: Impacts, Adaptation, and Vulnerability. Part A: Global and Sectoral Aspects. Contribution of Working Group II to the Fifth Assessment Report of the Intergovernmental Panel on Climate Change

[R37] Kaushal SS, Likens GE, Jaworski NA, Pace ML, Sides AM, Seekell D, Belt KT, Secor DH and Wingate RL 2010 Rising stream and river temperatures in the United States Front. Ecol. Environ 8 461–6

[R38] Kelleher C, Golden H and Archfield S HydroShare 2021 Data Release, US Stream Temperature Trend, HydroShare (10.4211/hs.b3773518e38b454780492d79e3a3f050)

[R39] Leach JA and Moore RD 2019 Empirical stream thermal sensitivities may underestimate stream temperature response to climate warming Water Resour. Res 55 5453–67

[R40] Lessard JL and Hayes DB 2003 Effects of elevated water temperature on fish and macroinvertebrate communities below small dams River Res. Appl 19 721–32

[R41] Mallakpour I and Villarini G 2017 Analysis of changes in the magnitude, frequency, and seasonality of heavy precipitation over the contiguous USA Theor. Appl. Climatol 130 345–63

[R42] Mann HB 1945 Nonparametric tests against trend Econometrica 13 245–59

[R43] Michel A, Brauchli T, Lehning M, Schaefli B and Huwald H 2020 Stream temperature and discharge evolution in Switzerland over the last 50 years: annual and seasonal behaviour Hydrol. Earth Syst. Sci 24 115–42

[R44] Mohseni O, Stefan HG and Erickson TR 1998 A nonlinear regression model for weekly stream temperatures Water Resour. Res 34 2685–92

[R45] Multi-Resolution Land Characteristics Consortium 2020 National Land Cover Database 2006 Land Cover (CONUS) (available at: www.mrlc.gov/data/nlcd-2006-land-cover-conus (Retrieved 4 May 2020))

[R46] Nayak MA, Herman JD and Steinschneider S 2018 Balancing flood risk and water supply in CA: policy search integrating short-term forecast ensembles with conjunctive use Water Resour. Res 54 7557–76

[R47] O’Keeffe JH, Palmer RW, Byren BA and Davies BR 1990 The effects of impoundment on the physicochemistry of two contrasting southern African river systems Regul. Rivers 5 97–110

[R48] Olden JD and Naiman RJ 2010 Incorporating thermal regimes into environmental flows assessments: modifying dam operations to restore freshwater ecosystem integrity Freshw. Biol 55 86–107

[R49] Piccolroaz S, Toffolon M, Robinson CT and Siviglia A 2018 Exploring and quantifying river thermal response to heatwaves Water 10 1098

[R50] Poole GC and Berman CH 2001 An ecological perspective on in-stream temperature: natural heat dynamics and mechanisms of human-caused thermal degradation Environ. Manage 27 787–802 11393314 10.1007/s002670010188

[R51] PRISM Climate Group 2004 Oregon State University (created 4 Feb 2004) (available at: http://prism.oregonstate.edu (Retrieved 4 November 2019))

[R52] Sleeter BM, Sohl TL, Loveland TR, Auch RF, Acevedo W, Drummond MA, Sayler KL and Stehman SV 2013 Land-cover change in the conterminous United States from 1973 to 2000 Glob. Environ. Change 23 733–48

[R53] Song C 2018 Continental-scale decrease in net primary productivity in streams due to climate warming Nat. Geosci 11 415–20

[R54] Steimke AL, Han B, Brandt JS and Flores AN 2018 Climate change and curtailment: evaluating water management practices in the context of changing runoff regimes in a snowmelt-dominated basin Water 10 1490

[R55] United States Army Corps 2019 National Inventory of Dams (available at: https://nid.sec.usace.army.mil/ords/f?p=105:19:16250180266113::NO::: (Retrieved 18 January 2021))

[R56] United States Geological Survey 2020 National Water Information System Data Available on the World Wide Web (USGS Water Data for the Nation (available at: 10.5066/F7P55KJN (Retrieved 5 March 2020))

[R57] United States Geological Survey 2021 StreamStats (v. 4.4.0) (available at: https://streamstats.usgs.gov/ss/ (Accessed 3 May 2020))

[R58] van Vliet MTH, Franssen WHP, Yearsley JR, Ludwig F, Haddeland I, Lettenmaier DP and Kabat P 2013 Global river discharge and water temperature under climate change Glob. Environ. Change 23 450–64

[R59] Wanders N 2019 High-resolution global water temperature modeling Water Resour. Res 55 2760–78

[R60] Webb BW 1996 Trends in stream and river temperature Hydrol. Process 10 205–26

[R61] Webb BW and Nobilis F 1997 Long-term perspective on the nature of the air–water temperature relationship: a case study Hydrol. Process 11 137–47

[R62] Wilde FD 2006 US Geological Survey Techniques of Water-Resources Investigations: Chapter A6. Section 6.1. Temperature (available at: 10.3133/twri09A6.1 (Accessed 2 August 2021))

[R63] Yan H, Sun N, Fullerton A and Baerwalde M 2021 Greater vulnerability of snowmelt-fed river thermal regimes to a warming climate Environ. Res. Lett 16 054006

