# Supplementary material for: Monthly river temperature trends across the US confound annual changes
Source: Environ Res Lett. Author manuscript; Available in PMC 2022 Dec 28. (PMC9797052; doi:10.1088/1748-9326/ac2289)
Supplement: SI [file NIHMS1746941-supplement-SI.docx]

**Supporting Information: Monthly River temperature Trends Across the US Confound Annual Responses**

Text S1: Classifying sites

We applied a classification scheme aimed at detecting dominant drivers of the thermal regime based on land cover and upstream distance to dams. Though the thermal regime is controlled by myriad factors, these dominant influences allowed us to create several classes that encompassed varying degrees of human impact.

We classified using six criteria:

*Classified as reference:* Falcone (2011) separated sites between reference (minimal human impact) and non-reference (human impact). We used this classification for any sites present within the GAGES II dataset. For sites outside of the GAGES II dataset

*Percentage of developed land cover:* sum of land cover classes 21, 22, 23, and 24 from the National Land Cover Database 2006 delineation (consistent with the GAGESII dataset)

*Percentage of agricultural land cover:* sum of land cover classes 81 and 82 from the National Land Cover Database 2006 delineation (consistent with the GAGES II dataset)

*Percentage of forested land cover:* sum of land cover classes 41, 42, and 43 from the National Land Cover Database 2006 delineation (consistent with the GAGES II dataset)

*Distance to nearest major dam:* raw straight-line distance to nearest major dam (height of 15.2 km or greater, and total storage of 6,167,400 m^3^; based on GAGES II definition) in watershed

*Presence of upstream dams:* presence of a dam of any size upstream.

Approximately 97 sites (20-year; 70% of sites) and 30 sites (40-year; 75% of sites) were present within the USGS GAGESII dataset; for these sites, the above criteria were extracted directly from the dataset. For sites that were not within this dataset, we first determined if these sites could be delineated within StreamStats. If delineation was possible, we used StreamStats watershed boundaries to extract land cover information. If delineation was not possible, we identified proximal sites within the GAGESII dataset, and used land cover information from these sites for classification. Regardless of delineation, all sites were screened to identify the distance to the nearest major dam within the watershed.

From this information, we applied the classification scheme described in section 2.1 of the main text to classify sites. The results of this classification are shown in Table S1.

Table S1: Number of sites with different classifications. Classifications were derived from existing USGS datasets (Dudley et al., 2018; Dudley et al., 2019) and information within the GAGESII dataset (Falcone et al., 2010; Falcone et al., 2011).

| Classification | 20-year trend (#) | 40-year trend (#) |
| --- | --- | --- |
| Agriculture | 2 | 0 |
| Agri-Urban | 1 | 0 |
| Compound stressors | 39 | 8 |
| Proximal regulation | 39 | 16 |
| Regulated | 39 | 11 |
| Reference | 17 | 5 |
| Unclassified | 1 | 0 |

Table S2: Positive annual trends, significant annual trends, significant positive annual trends, and significant annual negative trends for different periods, listed for number of sites and the percentage of total sites (20-year trend, n = 138 sites; 40-year trend, n = 40 sites).

| Trend Period and River temperature Observation | Sites with positive trend | | Sites with significant trends | | Significant positive trend | Significant negative trend |
| --- | --- | --- | --- | --- | --- | --- |
|  | Sen > 0 | | p-value < 0.05 | | Sen > 0;  p-value < 0.05 | Sen < 0;  p-value < 0.05 |
|  | Total | % | Total | % | Total | Total |
| 20 Year, Maximum  (n = 138) | 100 | 72.4 | 57 | 41.3 | 46 | 11 |
| 20 Year, Minimum  (n = 138) | 118 | 85.5 | 65 | 47.1 | 61 | 4 |
| 40 Year, Maximum  (n = 40) | 33 | 82.5 | 23 | 57.5 | 19 | 4 |
| 40 Year, Minimum  (n = 40) | 35 | 87.5 | 27 | 67.5 | 24 | 3 |

Table S3: Binned values (with a width of 0.05) for Mann Kendall tau for 20-year and 40-year maximum river temperature trends (Maximum) and minimum river temperature trends (Minimum). Values are expressed as a count of the 138 total sites (20-year) and 40 sites (40-year).

| Bin Center | 20-year | | 40-year | |  |
| --- | --- | --- | --- | --- | --- |
|  | Maximum (#) | Minimum (#) | Maximum (#) | Minimum (#) |  |
|  |  |  |  |  |  |
| -0.3 | 1 | 0 | 0 | 0 |  |
| -0.25 | 1 | 1 | 0 | 0 |  |
| -0.2 | 2 | 0 | 0 | 1 |  |
| -0.15 | 4 | 1 | 0 | 1 |  |
| -0.1 | 7 | 3 | 3 | 0 |  |
| -0.05 | 11 | 10 | 3 | 2 |  |
| 0 | 26 | 14 | 6 | 3 |  |
| 0.05 | 33 | 38 | 12 | 11 |  |
| 0.1 | 26 | 26 | 7 | 12 |  |
| 0.15 | 19 | 31 | 4 | 5 |  |
| 0.2 | 5 | 12 | 2 | 5 |  |
| 0.25 | 2 | 2 | 3 | 0 |  |
| 0.3 | 1 | 0 | 0 | 0 |  |

Table S4: 20-year trends in monthly-averaged daily maximum and minimum river temperatures, summarized as the number (#) and fraction (%) of sites with positive and negative trends during all months.

|  |  | Jan | Feb | Mar | Apr | May | Jun | Jul | Aug | Sep | Oct | Nov | Dec |
| --- | --- | --- | --- | --- | --- | --- | --- | --- | --- | --- | --- | --- | --- |
| Maximum | Sen > 0,  Sites (#) | 88 | 98 | 91 | 64 | 89 | 103 | 94 | 64 | 96 | 101 | 55 | 83 |
|  | Sen < 0,  Sites (#) | 47 | 38 | 44 | 74 | 48 | 35 | 44 | 73 | 42 | 37 | 83 | 54 |
|  | Sen > 0,  Sites (%) | 63.8 | 71.0 | 65.9 | 46.4 | 64.5 | 74.6 | 68.1 | 46.4 | 69.6 | 73.2 | 39.9 | 60.1 |
|  | Sen < 0,  Sites (%) | 34.1 | 27.5 | 31.9 | 53.6 | 34.8 | 25.4 | 31.9 | 52.9 | 30.4 | 26.8 | 60.1 | 39.1 |
| Minimum | Sen > 0,  Sites (#) | 91 | 107 | 115 | 84 | 95 | 106 | 108 | 80 | 105 | 115 | 57 | 88 |
|  | Sen < 0,  Sites (#) | 40 | 27 | 21 | 54 | 43 | 32 | 30 | 58 | 32 | 23 | 80 | 47 |
|  | Sen > 0,  Sites (%) | 65.9 | 77.5 | 83.3 | 60.9 | 68.8 | 76.8 | 78.3 | 58.0 | 76.1 | 83.3 | 41.3 | 63.8 |
|  | Sen < 0,  Sites (%) | 29.0 | 19.6 | 15.2 | 39.1 | 31.2 | 23.2 | 21.7 | 42.0 | 23.2 | 16.7 | 58.0 | 34.1 |

Table S5: Percentage of sites with significant (p-value < 0.05), significant positive (Sen > 0), and significant negative (Sen < 0) 20-year trends in monthly-averaged daily maximum and minimum river temperatures across all months.

|  |  | Jan | Feb | Mar | Apr | May | Jun | Jul | Aug | Sep | Oct | Nov | Dec |
| --- | --- | --- | --- | --- | --- | --- | --- | --- | --- | --- | --- | --- | --- |
| Maximum | Significant | 2.9 | 4.3 | 2.2 | 3.6 | 9.4 | 13.8 | 14.5 | 10.9 | 15.9 | 11.6 | 3.6 | 3.6 |
|  | & Sen > 0 | 0.0 | 1.4 | 2.2 | 2.2 | 7.2 | 9.4 | 10.1 | 4.3 | 12.3 | 10.1 | 1.4 | 2.2 |
|  | & Sen < 0 | 2.9 | 2.9 | 0.0 | 1.4 | 2.2 | 4.3 | 4.3 | 6.5 | 3.6 | 1.4 | 2.2 | 1.4 |
| Minimum | Significant | 2.9 | 8.0 | 2.9 | 4.3 | 7.2 | 15.9 | 12.3 | 13.0 | 21.0 | 15.9 | 4.3 | 4.3 |
|  | Sen > 0 | 0.0 | 6.5 | 2.2 | 3.6 | 6.5 | 13.0 | 11.6 | 9.4 | 18.1 | 14.5 | 2.2 | 3.6 |
|  | Sen < 0 | 1.4 | 0.7 | 0.7 | 0.7 | 0.7 | 2.9 | 0.7 | 3.6 | 2.9 | 1.4 | 2.2 | 0.7 |

Table S6: 40-year trends in monthly-averaged daily maximum and minimum river temperatures, summarized as the number (#) and fraction (%) of sites with positive and negative trends during all months.

|  |  | Jan | Feb | Mar | Apr | May | Jun | Jul | Aug | Sep | Oct | Nov | Dec |
| --- | --- | --- | --- | --- | --- | --- | --- | --- | --- | --- | --- | --- | --- |
| Maximum | Sen > 0,  Sites (#) | 37 | 36 | 28 | 25 | 22 | 23 | 27 | 22 | 23 | 25 | 27 | 26 |
|  | Sen < 0,  Sites (#) | 3 | 4 | 12 | 15 | 18 | 17 | 13 | 17 | 17 | 15 | 13 | 13 |
|  | Sen > 0,  Sites (%) | 92.5 | 90.0 | 70.0 | 62.5 | 55.0 | 57.5 | 67.5 | 55.0 | 57.5 | 62.5 | 67.5 | 65.0 |
|  | Sen < 0,  Sites (%) | 7.5 | 10.0 | 30.0 | 37.5 | 45.0 | 42.5 | 32.5 | 42.5 | 42.5 | 37.5 | 32.5 | 32.5 |
| Minimum | Sen > 0,  Sites (#) | 37 | 37 | 29 | 30 | 28 | 27 | 28 | 26 | 25 | 26 | 25 | 28 |
|  | Sen < 0,  Sites (#) | 2 | 3 | 11 | 10 | 12 | 13 | 12 | 14 | 15 | 14 | 15 | 12 |
|  | Sen > 0,  Sites (%) | 92.5 | 92.5 | 72.5 | 75.0 | 70.0 | 67.5 | 70.0 | 65.0 | 62.5 | 65.0 | 62.5 | 70.0 |
|  | Sen < 0,  Sites (%) | 5.0 | 7.5 | 27.5 | 25.0 | 30.0 | 32.5 | 30.0 | 35.0 | 37.5 | 35.0 | 37.5 | 30.0 |

Table S7: Number of sites annually warming but with monthly cooling for between 0 and 8+ months. Values are shown for monthly-averaged daily maximum and daily minimum river temperature twenty-year and forty-year trends.

| Trend Period | River temperature value | Total | 0 | 1 | 2 | 3 | 4 | 5 | 6 | 7 | 8+ |
| --- | --- | --- | --- | --- | --- | --- | --- | --- | --- | --- | --- |
| 20-year | Maximum | 100 | 2 | 14 | 18 | 25 | 13 | 19 | 7 | 2 | 0 |
| 20-year | Minimum | 118 | 6 | 22 | 26 | 25 | 13 | 16 | 6 | 4 | 0 |
| 40-year | Maximum | 33 | 2 | 8 | 1 | 7 | 7 | 4 | 3 | 0 | 1 |
| 40-year | Minimum | 35 | 3 | 10 | 1 | 11 | 5 | 2 | 3 | 0 | 0 |

Table S8: Comparison of air and river temperature trends. Percentage of sites where differences between air and river temperature trends are near zero (difference of -0.1 to 0.1°C 10 yrs^-1^), where trends in river temperature exceed air temperature (difference > 0.1°C 10 yrs^-1^), and where trends in air temperature exceed river temperature (difference < -0.1°C 10 yrs^-1^).

| Season | Month | Difference between Monthly-Averaged Maximum Air (AT) and River (RT) temperature Trends  (°C 10 yrs^-1^) | | | Difference between Monthly-Averaged Minimum Air (AT) and River (RT) temperature Trends  (°C 10 yrs^-1^) | | |
| --- | --- | --- | --- | --- | --- | --- | --- |
|  |  | < -0.1  (WT < AT) | -0.1 to 0.1 | > 0.1  (WT > AT) | < -0.1  (WT < AT) | -0.1 to 0.1 | > 0.1  (WT > AT) |
| Winter | December | 64.5 | 8.7 | 26.8 | 45.7 | 7.2 | 47.1 |
|  | January | 56.5 | 4.3 | 39.1 | 57.2 | 15.2 | 26.1 |
|  | February | 67.4 | 10.9 | 21.7 | 47.1 | 13.8 | 39.1 |
| Spring | March | 61.6 | 13.0 | 25.4 | 36.2 | 19.6 | 44.2 |
|  | April | 51.4 | 13.8 | 34.8 | 32.6 | 16.7 | 50.7 |
|  | May | 26.1 | 10.1 | 63.8 | 13.0 | 11.6 | 75.4 |
| Summer | June | 44.2 | 8.7 | 47.1 | 31.9 | 16.7 | 51.4 |
|  | July | 42.8 | 9.4 | 47.8 | 31.9 | 15.2 | 52.9 |
|  | August | 39.9 | 15.2 | 44.9 | 21.7 | 14.5 | 63.8 |
| Fall | September | 47.1 | 19.6 | 32.6 | 9.4 | 9.4 | 81.2 |
|  | October | 63.8 | 10.1 | 26.1 | 17.4 | 10.9 | 71.7 |
|  | November | 64.5 | 8.7 | 26.8 | 55.1 | 13.8 | 31.2 |

Table S9: Linear (r^2^) and nonlinear (Spearman’s Rho, $\rho$) relationships between 20-year trends (annual and monthly) with drainage area and latitude. Drainage area was not able to be defined for 4 sites.

| Period | Daily Maximum | | | | Daily Minimum | | | |
| --- | --- | --- | --- | --- | --- | --- | --- | --- |
|  | Drainage Area | | Latitude | | Drainage Area | | Latitude | |
|  | r^2^ | $\rho$ | r^2^ | $\rho$ | r^2^ | $\rho$ | r^2^ | $\rho$ |
| Annual | 0.01 | -0.33 | 0.00 | -0.18 | 0.01 | -0.31 | 0.00 | -0.13 |
| January | 0.02 | -0.11 | 0.01 | -0.24 | 0.02 | -0.03 | 0.03 | -0.25 |
| February | 0.00 | -0.09 | 0.00 | -0.15 | 0.00 | -0.02 | 0.01 | -0.22 |
| March | 0.00 | -0.06 | 0.01 | -0.16 | 0.01 | -0.12 | 0.01 | -0.15 |
| April | 0.00 | -0.23 | 0.00 | -0.05 | 0.00 | -0.24 | 0.00 | 0.00 |
| May | 0.02 | -0.36 | 0.06 | 0.23 | 0.02 | -0.34 | 0.03 | 0.16 |
| June | 0.01 | -0.14 | 0.01 | 0.05 | 0.01 | -0.08 | 0.00 | -0.02 |
| July | 0.03 | -0.16 | 0.01 | -0.01 | 0.04 | -0.13 | 0.00 | -0.04 |
| August | 0.00 | -0.15 | 0.06 | 0.17 | 0.00 | -0.21 | 0.06 | 0.18 |
| September | 0.00 | 0.02 | 0.03 | -0.25 | 0.00 | 0.08 | 0.02 | -0.25 |
| October | 0.00 | -0.08 | 0.01 | -0.17 | 0.00 | -0.10 | 0.02 | -0.19 |
| November | 0.01 | -0.15 | 0.10 | 0.30 | 0.01 | -0.11 | 0.09 | 0.29 |
| December | 0.00 | -0.07 | 0.14 | -0.37 | 0.00 | 0.02 | 0.20 | -0.42 |

Table S10: Linear (r^2^) and nonlinear (Spearman’s Rho, $\rho$) relationships between 40-year (n = 40 sites) trends (annual and monthly) with drainage area and latitude.

| Period | Daily Maximum | | | | Daily Minimum | | | |
| --- | --- | --- | --- | --- | --- | --- | --- | --- |
|  | Drainage Area | | Latitude | | Drainage Area | | Latitude | |
|  | r^2^ | $\rho$ | r^2^ | $\rho$ | r^2^ | $\rho$ | r^2^ | $\rho$ |
| Annual | 0.00 | -0.09 | 0.00 | 0.08 | 0.01 | -0.11 | 0.00 | -0.08 |
| January | 0.01 | -0.36 | 0.00 | 0.01 | 0.01 | -0.38 | 0.00 | -0.06 |
| February | 0.00 | -0.10 | 0.01 | -0.02 | 0.00 | -0.13 | 0.04 | -0.11 |
| March | 0.00 | 0.28 | 0.09 | -0.31 | 0.00 | 0.17 | 0.09 | -0.35 |
| April | 0.01 | -0.06 | 0.00 | -0.06 | 0.03 | -0.05 | 0.01 | -0.14 |
| May | 0.00 | -0.10 | 0.01 | 0.12 | 0.02 | -0.18 | 0.00 | 0.05 |
| June | 0.00 | 0.05 | 0.02 | 0.19 | 0.00 | 0.08 | 0.01 | 0.10 |
| July | 0.01 | 0.06 | 0.03 | 0.34 | 0.00 | 0.08 | 0.04 | 0.40 |
| August | 0.00 | 0.06 | 0.00 | 0.05 | 0.00 | 0.04 | 0.00 | 0.13 |
| September | 0.00 | 0.06 | 0.02 | 0.21 | 0.00 | 0.13 | 0.01 | 0.10 |
| October | 0.01 | -0.06 | 0.01 | -0.07 | 0.01 | -0.06 | 0.02 | -0.20 |
| November | 0.02 | -0.20 | 0.00 | -0.14 | 0.01 | -0.07 | 0.00 | -0.15 |
| December | 0.05 | -0.31 | 0.00 | -0.13 | 0.05 | -0.30 | 0.02 | -0.22 |


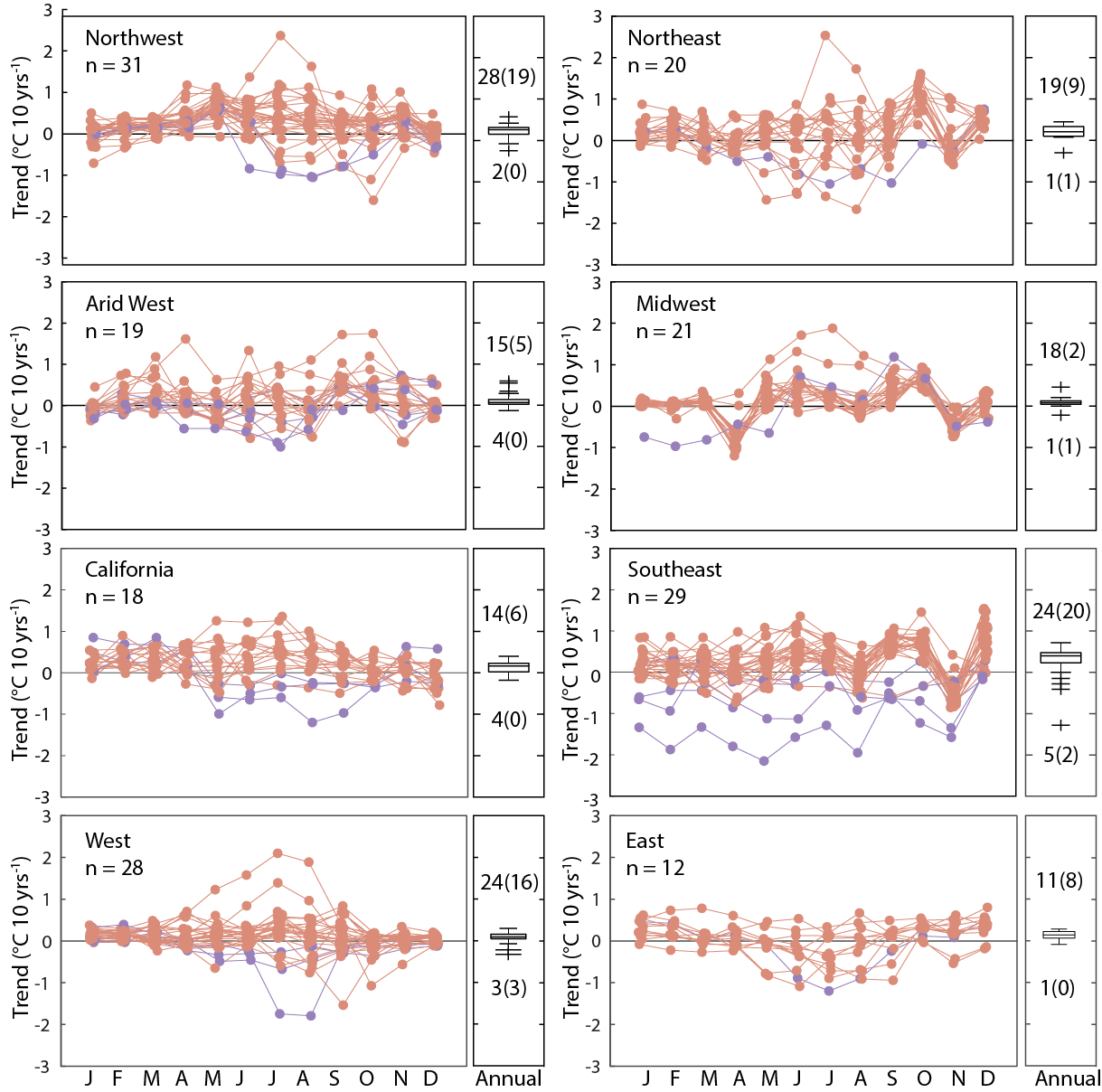


Figure S1: Annual and monthly (a) 20-year and (b) 40-year trends in monthly-averaged minimum daily stream temperatures are shown separated by region. Monthly trends are shown as line plots, while annual trends are aggregated into box plots. Line colors indicate whether annual trends were positive (red) or negative (blue). Numbers indicate the number of positive and negative annual trends, while numbers in parentheses indicate the number of significant positive or negative annual trends. Note that three sites (20-year) and one site (40-year) had an annual trend of zero.

**
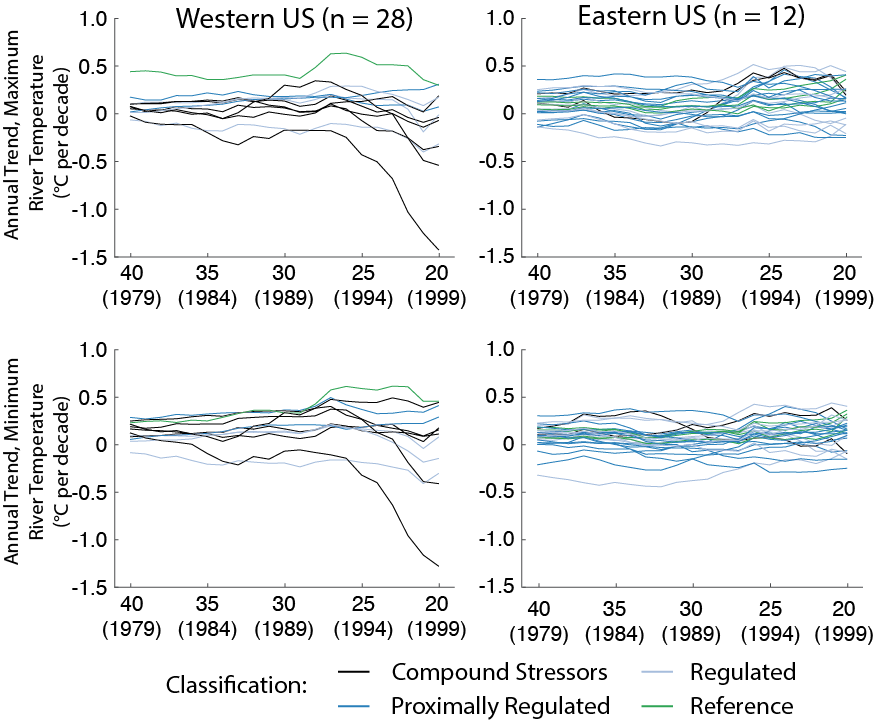
**

Figure S2: River temperature trend trajectories. Sen slopes for annual trends assessed via the seasonal MK trend test are shown for 20-year (1999) to 40-year (1979) periods across sites with long term observations. Sites span the western (left) and eastern US (right) and are shown for both monthly-averaged daily maximum (top) and minimum (bottom) river temperature. Each line corresponds to a different site, with color indicating site classification.


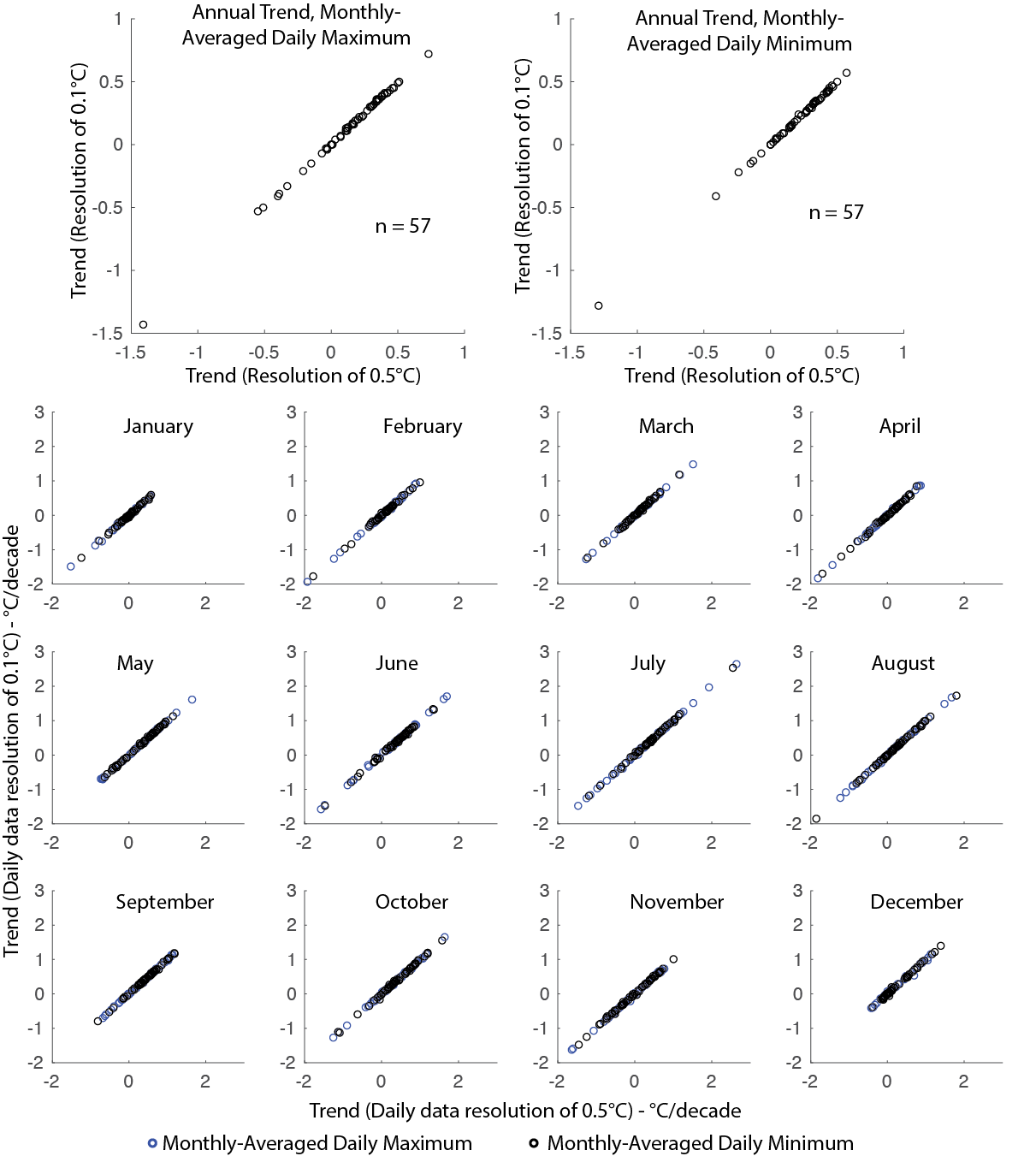


Figure S3: Annual and monthly 20-year trends computed with high resolution observations (daily river temperatures reported to resolution of 0.1°C) versus trends computed rounding daily river temperatures to a lower resolution (0.5°C). Trends were computed for 57 sites with high resolution observations for the period of record (1999 – 2018).


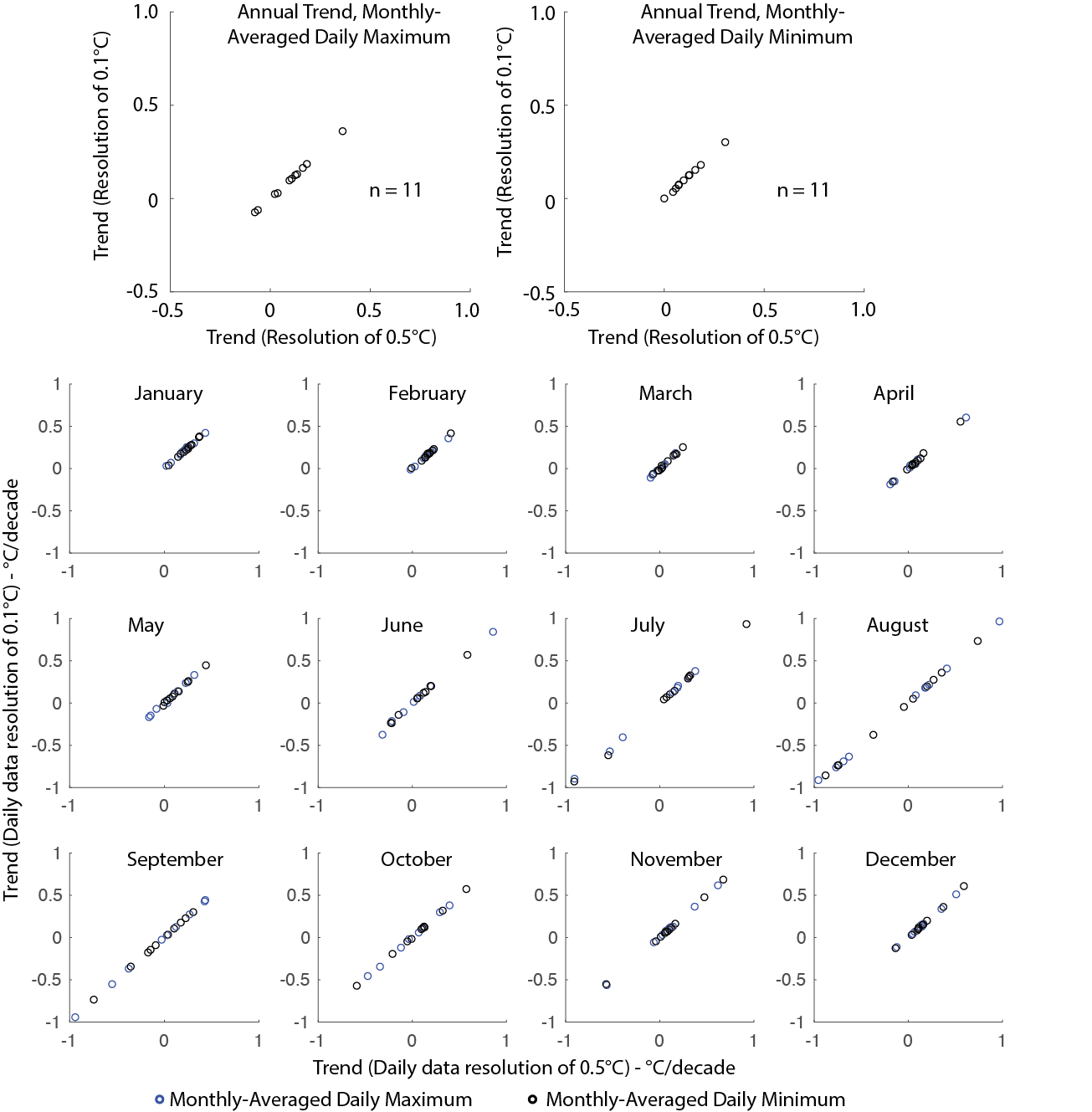


Figure S4: Annual and monthly 40-year trends computed with high resolution observations (daily river temperatures reported to resolution of 0.1°C) versus trends computed rounding daily river temperatures to a lower resolution (0.5°C). Trends were computed for 11 sites with high resolution observations for the period of record (1999 – 2018).


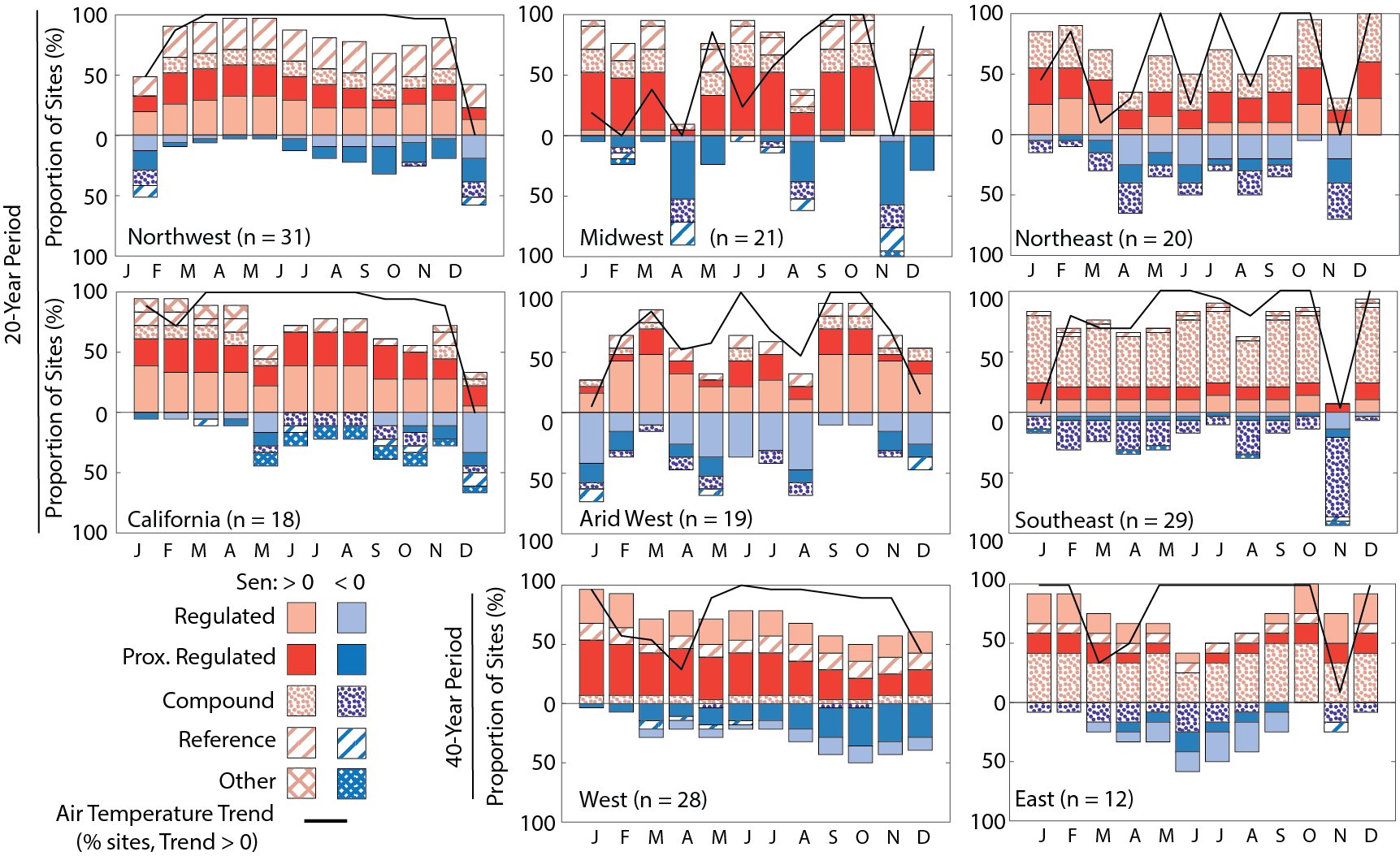
Figure S5: Monthly 20-year (top) and 40-year (bottom) trends for monthly-averaged daily minimum river temperatures (determined via MK) showing the number of sites with positive (Sen > 0) or negative (Sen < 0) trends. While 20-year trends span the conterminous US (Fig. 1), 40-year trends were only concentrated in the west and eastern US. Black lines indicate the proportion of sites with positive monthly air temperature trends. Fill indicates site classification. Results for monthly-averaged minimum river temperature trends are shown in Figure S2. Class ‘Other’ compasses one Agri-Urban site, two Agriculture sites, and one Unclassified site.


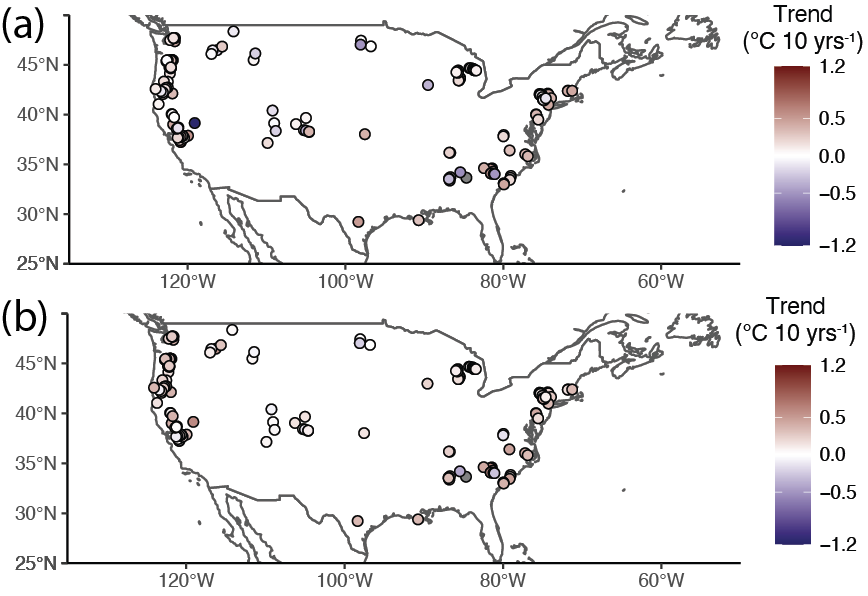


Figure S6: River temperature trend magnitudes (20-year period) for (a) monthly-averaged maximum daily river temperature and (b) monthly-averaged minimum daily river temperature.


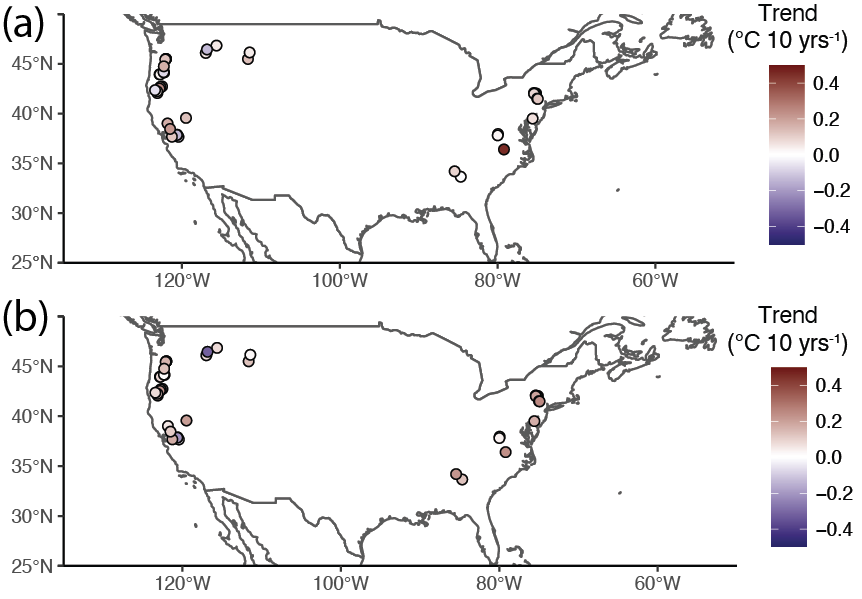


Figure S7: River temperature trend magnitudes (40-year period) for (a) monthly-averaged maximum daily river temperature and (b) monthly-averaged minimum daily river temperature.


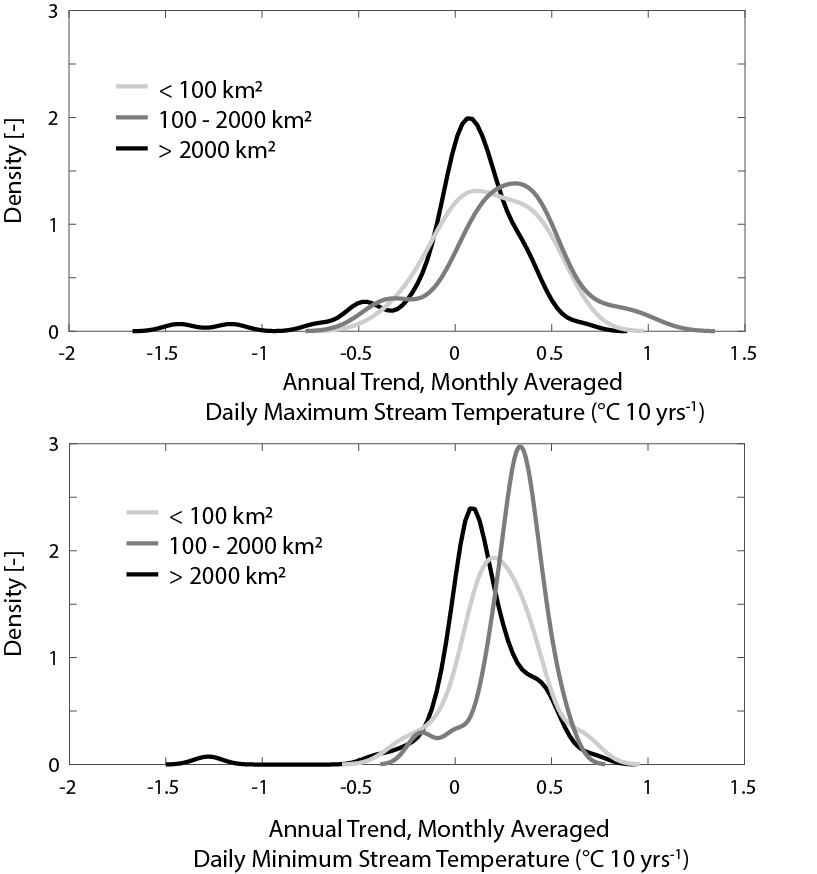


Figure S8: Probability density plots of annual trends separated by site drainage area. Results are shown for 20-year monthly-averaged daily maximum (top) and monthly-averaged daily minimum (bottom) trends.
